# Decision Making Impairment: A Shared Vulnerability in Obesity, Gambling Disorder and Substance Use Disorders?

**DOI:** 10.1371/journal.pone.0163901

**Published:** 2016-09-30

**Authors:** Nuria Mallorquí-Bagué, Ana B. Fagundo, Susana Jimenez-Murcia, Rafael de la Torre, Rosa M. Baños, Cristina Botella, Felipe F. Casanueva, Ana B. Crujeiras, Jose C. Fernández-García, Jose M. Fernández-Real, Gema Frühbeck, Roser Granero, Amaia Rodríguez, Iris Tolosa-Sola, Francisco J. Ortega, Francisco J. Tinahones, Eva Alvarez-Moya, Cristian Ochoa, Jose M. Menchón, Fernando Fernández-Aranda

**Affiliations:** 1 Department of Psychiatry, University Hospital of Bellvitge-IDIBELL, Barcelona, Spain; 2 CIBER Fisiopatología de la Obesidad y Nutrición (CIBERobn), Instituto Salud Carlos III, Madrid, Spain; 3 Department of Clinical Sciences, School of Medicine, University of Barcelona, Barcelona, Spain; 4 Integrated Pharmacology and Systems Neurosciences Research Group, Neuroscience Research Program, IMIM (Hospital del Mar Medical Research Institute), Barcelona, Spain, Pompeu Fabra University (CEXS-UPF), Barcelona, Spain; 5 Department of Psychological, Personality, Evaluation and Treatment of the University of Valencia, Valencia, Spain; 6 Department of Basic Psychology, Clinic and Psychobiology of the University Jaume I, Castelló, Spain; 7 Endocrine Division, Complejo Hospitalario U. de Santiago, Santiago de Compostela University, Santiago de Compostela, Spain; 8 Department of Endocrinology and Nutrition, Hospital Clínico Universitario Virgen de Victoria, Málaga, Spain; 9 Department of Diabetes, Endocrinology and Nutrition, Institut d’Investigació Biomèdica de Girona (IdlBGi) Hospital Dr Josep Trueta, Girona, Spain; 10 Department of Endocrinology and Nutrition, Clínica Universidad de Navarra, University of Navarra, Pamplona, Spain; 11 Departament de Psicobiologia i Metodologia, Universitat Autònoma de Barcelona, Barcelona, Spain; 12 CIBER Salud Mental (CIBERsam), Instituto Salud Carlos III, Madrid, Spain; Ariel University, ISRAEL

## Abstract

**Introduction:**

Addictions are associated with decision making impairments. The present study explores decision making in Substance use disorder (SUD), Gambling disorder (GD) and Obesity (OB) when assessed by Iowa Gambling Task (IGT) and compares them with healthy controls (HC).

**Methods:**

For the aims of this study, 591 participants (194 HC, 178 GD, 113 OB, 106 SUD) were assessed according to DSM criteria, completed a sociodemographic interview and conducted the IGT.

**Results:**

SUD, GD and OB present impaired decision making when compared to the HC in the overall task and task learning, however no differences are found for the overall performance in the IGT among the clinical groups. Results also reveal some specific learning across the task patterns within the clinical groups: OB maintains negative scores until the third set where learning starts but with a less extend to HC, SUD presents an early learning followed by a progressive although slow improvement and GD presents more random choices with no learning.

**Conclusions:**

Decision making impairments are present in the studied clinical samples and they display individual differences in the task learning. Results can help understanding the underlying mechanisms of OB and addiction behaviors as well as improve current clinical treatments.

## Introduction

Evidence based neurocognitive models of addiction propose that addiction-related behaviors are the result of an imbalance of three neural systems: an impulsive neural system that promotes habitual and salient behaviors, interoceptive processes that are involved in uncertain risk and reward, and a reflective neural system for inhibitory control and decision-making [1][2]. Decision-making entails the cognitive process of making a choice after reflection on the consequences of that choice, and it is a key component of addiction development and maintenance in both substance use disorders (SUD) and behavioral addictions such as gambling disorder (GD). The assessment of decision-making is usually conducted through the Iowa Gambling Task (IGT), which simulates real life decision making strategies by factoring uncertainty, reward and punishment. The IGT is a relatively complex task as it is difficult to deconstruct into different cognitive constructs, however it measures decision-making with a high ecological validity[3] in a wide range of clinical and non-clinical groups[4]. Specifically, impairments in this task have been demonstrated in patients with ventromedial prefrontal cortex lesions (VMPC;[5]) and in different psychopathological conditions including addictions and eating disorders[6–8]. Distinctively, while clinical individuals with decision making impairment fail to learn the contingencies and prefer the choices that lead to higher long term losses, healthy individuals present a gradual learning across the IGT [4][5]. According to the Somatic Marker Hypothesis, individuals who perform poorly on the IGT have weaker physiological cues to guide risky choices and present what is referred to as “myopia for the future” [9].

Complementary, obesity (OB) is an increasing worldwide problem that shares similar patterns to addictions[6]. Individuals with obesity frequently decide to overeat despite being aware of its negative long-term health consequences and they usually put extra but unsuccessful efforts into controlling their eating behaviour. Neuropsychological studies support the hypothesis of an alteration on inhibitory control, emotion regulation and the executive function circuit in which one of the core cognitive traits appears to be decision making[10]. Accordingly, recent data shows that individuals with obesity are characterized by the tendency to engage in decisions that support a positive short-term reword even if it results in long term negative outcomes[7]. Furthermore when assessed by the IGT, individuals with obesity present significant decision making impairments in the overall task performance as well as in learning across the task [7,11,12]. Likewise GD and SUD individuals present a similar decision making pattern, being the overall task performance and learning across the task impaired[8]. For instance, a recent study conducted with GD participants describes a strong preference for choices featuring high rewards rather than higher losses during the IGT. The authors of this study suggest that this might reflect an hypersensitivity of their reward systems[13]. Additionally, GD and SUD are usually associated; being the decision-making patterns worse when both diagnoses are present[14]. Similarly, OB and GD display neurocognitive and clinical associations. For instance OB is associated with decision making and sustained attention impairments in gamblers, along with greater monetary losses due to gambling [15].

Direct comparisons of decision making profiles among SUD, GD and OB groups have yet to be conducted. However, direct comparisons can provide a valuable insight into the similarities and/or singularities of different addictive related behaviours.

### Aims of the study

The present study aims to further explore the decision making profiles of Substance use disorder, Gambling disorder and Obesity when assessed by the Iowa gambling task and compares them with healthy controls (HC). The specific aims of the study are the following: (1) compare the overall performance of the three clinical groups and the healthy controls, (2) observe and compare specific patterns of learning across the task in the three clinical conditions and the healthy controls. It is hypothesized that the clinical samples will present poorer performances on the IGT when compared to the HC group. Also, specific patterns of learning across the task will be observed in the four studied samples. The results have the potential of improving our understanding of the specific executive profiles (namely decision making) underlying the association between obesity and addictive behaviours which in turn can also help improving current obesity treatments.

## Methods

### Sample

The final sample consists of 591 participants (51.7% females) distributed as follow: 194 HC, 178 GD, 113 OB and 106 SUD individuals. GD and SUD diagnostic criterion were assessed by an experienced clinician (according to the DSM-IV-TR), by means of SCID-I [16].

Seven centers, all involved in the CIBERobn Spanish Research Network, participated in the study: the Eating Disorders Unit (Department of Psychiatry, University Hospital of Bellvitge-IDIBELL, Barcelona), the Department of Endocrinology (University Hospital of Santiago, Santiago de Compostela); the Department of Diabetes, Endocrinology and Nutrition (Clinic University Hospital Virgen de Victoria, Malaga); the Department of Endocrinology and Nutrition (University of Navarra, Pamplona); the Diabetes, Endocrinology and Nutrition Department, (Biomedical Research Institute of Girona IdIBGi-Doctor Josep Trueta Hospital, Girona); the Clinical Research Unit (Hospital del Mar Medical Research Institute-IMIM, Barcelona) and the Department of Basic Psychology, Clinic and Psychobiology (University Jaume I, Castellón). The GD, SUD and OB participants were patients that were consecutively referred to the clinics mentioned above. Recruitment of the controls took place by means of word-of-mouth and advertisements at local universities.. All participants gave written signed informed consent and received no additional compensation for being part of the study. In accordance with the Helsinki Declaration of 1975 as revised in 1983, the Ethics Committee of all the institutions involved in the project approved the study: Comissió Deontológica de la Universitat Jaume I, Subcomisión Clínica del Hospital Universitario “Virgen de la Victoria”, Málaga, Comite Etic de Investigacio Clinica Hospital Universitari de Girona Doctor Josep Trueta (048/10), Comite Etico de Investigacion Clinica del Consorci Mar Parc de Salut de Barcelona-Parc de Salut Mar (2010/3914/I), Comité de Etica de la Investigación Universidad de Navarra (110/2010) and Comité Etico de Investigación Clínica del Hospital Universitari de Bellvitge (307/06)]. Exclusion criterion were: (1) history of chronic medical illness or neurological condition that might affect cognitive function; (2) head trauma, learning disability or intellectual disabilities; (3) individuals who have suffered a lifetime mental disorder according to DSM-IV-TR other than the specific disorder of study (including the following: no OB individual had a lifetime eating disorder, GD participants had no SUD, no SUD individual had GD); (4) age under 18 or over 65 (to discard neuropsychological deficits associated with age); (5) having diabetes mellitus. There was one extra exclusion criteria for all groups except from the SUD group: (6) history of substance abuse in the previous 3 months and use of psychoactive medication or drugs. The SUD individuals had not taken any drugs during the last 72 hours prior to explorations (assessed by urine drug testing). Finally, in addition to the already mentioned criterion, for the OB group (7) patients with obesity who had comorbid binge eating disorder (DSM-IV-TR) were also excluded.

### Neuropsychological assessment

For the purpose of this study all individuals were assessed with the IGT [4]. This computer task evaluates decision-making, risk and reward as well as punishment value. The subject has to select 100 cards from four decks (A, B, C and D). After each card selection an output is given: gain or a gain and loss of money. Two decks (A and B) are not advantageous as the final loss is higher than the final gain. Decks C and D, however, are advantageous since the punishments are smaller. The final objective of the task is to win as much money as possible. Before completing the task, all participants were instructed to try to win as much money as possible and avoid losing as much as money as possible and, they were also informed that some decks were worse than others. This test is scored by subtracting the amount of cards selected from decks A and B from the amount of cards selected from decks C and D. It provides information about task learning (NET 1 to 5) as well as an overall performance score (NET_Total).

### Statistical analysis

Analyses were carried out with SPSS20 for Windows. Chi-square (χ^2^) tests compared categorical variables between the diagnostic subtypes. Analysis of Variance (ANOVA), adjusted by the covariates age and years of education, compared the means for the cognitive measures (IGT scores). The ANOVA procedures included the between-subjects factors sex (two levels: men versus women) and diagnostic subtype (four levels: HC, GD, OB and SUD), as well as the interaction group-by-sex to explore the potential moderator effect of sex into the relationships between diagnosis and cognition outcomes. The Holm-Bonferroni method, which is one of the Familywise error rate stepwise procedures that offers more powerful tests than the classical Bonferroni-correction, was used to control Type-I error due to multiple comparisons[17].

## Results

Table 1 includes the results of the χ^2^ tests and the ANOVA to compare the sociodemographic variables between groups. Descriptive variables of our sample (Table 1) show the inherent differences in sex, age and education among clinical groups (GD, SUD and OB groups). Accordingly and for the statistical aims of this study we controlled for the age and education level variables and used them as covariates in the ANOVA when comparing IGT scores.

**Table 1:** Sociodemographic descriptive.

	HC		GD		OB		SUD		Factor: group			Pairwise comparisons: p-value					
	n = 194		n = 178		n = 113		n = 106		^1^Stat	df	p	HC-GD	HC-OB	HC-SUD	GD-OB	GD-SUD	OB-SUD
**Sex; n(%) Females**	154	79.4%	17	9.6%	87	77.0%	32	30.2%	232.9	3	< .001	< .001	.623	< .001	< .001	< .001	< .001
**Males**	40	20.6%	161	90.4%	26	23.0%	74	69.8%									
**Civil status; n(%) Single**	155	79.9%	26	14.6%	13	11.5%	---	---	217.7	4	< .001	< .001	< .001	---	.269	---	---
**Married—in couple**	35	18.0%	144	80.9%	90	79.6%	---	---									
**Divorced—separated**	4	2.1%	8	4.5%	10	8.8%	---	---									
**Employment; n(%) Unemployed**	133	68.6%	6	3.4%	16	14.2%	---	---	202.9	2	< .001	< .001	< .001	---	.001	---	---
**Employed**	61	31.4%	172	96.6%	97	85.8%	---	---									
**Education; n(%) Primary**	56	28.9%	156	87.6%	19	16.8%	77	72.6%	243.9	6	< .001	< .001	< .001	< .001	< .001	< .001	< .001
**Secondary**	81	41.8%	18	10.1%	78	69.0%	8	7.5%									
**University**	57	29.4%	4	2.2%	16	14.2%	21	19.8%									
**Age (years-old); Mean-SD**	24.95	7.10	38.28	10.94	43.38	10.42	21.66	2.87	189.9	3;587	< .001	< .001	< .001	.002	< .001	< .001	< .001
**Education (yrs); Mean-SD**	14.53	3.73	12.17	2.89	12.87	3.95	11.69	2.85	21.8	3;587	< .001	< .001	< .001	< .001	.090	.245	.011

SD: standard deviation.

^1^Statistic: χ2-statistic for proportion comparison and F-statistic for mean comparison.

--- Not available for this group. HC: healthy control. GD: gambling disorder. SUD: substance use disorder. OB: obesity.

**Fig 1:**
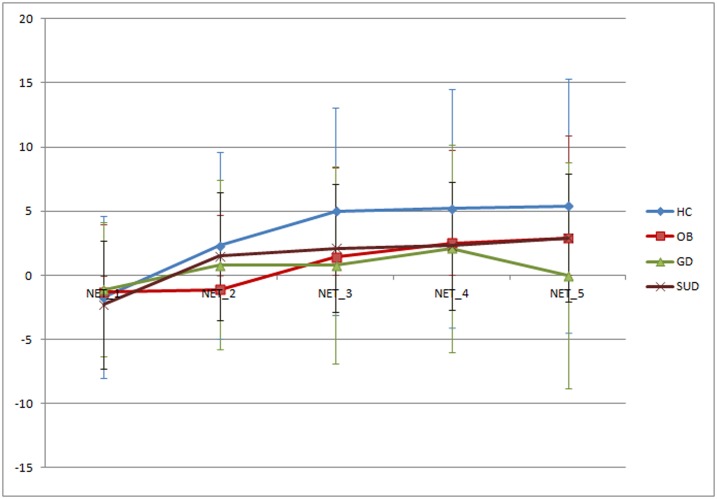
Mean cognitive measures in learning across the task between groups.

Table 2 includes the ANOVA model comparing the cognitive mean scores measured through the IGT, adjusted by the covariates age and years of education. The first step of the ANOVA procedure tested the interaction parameter ‘group-by-sex’. The absence of significant moderation effects indicates no sex significant differences in the diagnostic subtypes and it also indicates that differences between sexes were statistically equal among the diagnostic subtypes. Consequently, the interaction parameter term was excluded from the modeling and the main effects for the factors group and sex were obtained and interpreted. Regarding the group factor analysis, differences among groups emerged in all NET scores but in NET-1. Additionally, HC participants achieved higher mean scores in NET-total, NET-3 and NET-4 when compared to the other clinical conditions (GD, OB and SUD). The HC participants also achieved a higher mean score in NET-5 when compared to GD and SUD individuals and, GD participants obtained a lower mean score compared to OB and SUD individuals. Concerning NET-2, the OB group achieved the lowest mean score and it was statistically different when compared to the other clinical conditions. Finally, with reference to the sex factor analysis, differences between men and women emerged in all IGT scores except in NET-1 and NET-2 ones. Specifically, men achieved significantly higher means than women. Fig 1, displays the comparison of the mean scores in the cognitive learning across the task scales between groups.

**Table 2:** Comparison of the cognitive mean scores between diagnostic subtypes: ANOVA.

	Interaction		HC		GD		OB		SUD		Factor		Pairwise comparisons												Women		Men		Factor		
	group×sex		*n* = 194		*n* = 178		*n* = 113		*n* = 106		group		HC vs GD		HC vs OB		HC vs SUD		GD vs OB		GD vs SUD		OB vs SUD		*n* = 290		*n* = 301		Sex		
	F_3;581_	*p*	Mean	SD	Mean	SD	Mean	SD	Mean	SD	F_3;584_	*p*	MD	*p*	MD	*p*	MD	*p*	MD	*p*	MD	*p*	MD	*p*	Mean	SD	Mean	SD	F_1;584_	*p*	MD
NET_total	0.94	.419	16.07	27.7	2.14	23.8	4.33	22.8	6.45	21.7	6.51	**.001**	13.93	**.001**	11.74	**.001**	9.61	**.003**	-2.19	.518	-4.31	.231	-2.13	.615	3.02	26.3	11.48	23.8	10.76	**.001**	8.45
NET_1	0.28	.842	-1.69	6.3	-1.15	5.2	-1.25	5.3	-2.30	4.3	0.69	.559	-0.55	.493	-0.44	.582	0.61	.410	0.10	.895	1.15	.159	1.05	.272	-1.62	6.0	-1.58	5.0	0.01	.953	-0.03
NET_2	0.24	.868	2.25	7.3	0.83	6.6	-1.11	5.8	1.51	5.5	4.30	**.016**	1.42	.128	3.35	**.001**	0.74	.387	1.93	**.032**	-0.68	.479	-2.61	**.020**	0.53	7.0	1.21	6.2	0.97	.326	-0.67
NET_3	0.36	.784	5.00	8.1	0.80	7.7	1.39	7.0	2.05	6.3	6.42	**.001**	4.20	**.001**	3.61	**.001**	2.95	**.003**	-0.59	.569	-1.25	.258	-0.66	.611	1.51	7.7	3.11	7.5	4.07	**.044**	-1.59
NET_4	1.61	.185	5.17	9.3	2.12	8.1	2.50	7.3	2.23	7.2	3.56	**.028**	3.05	**.010**	2.67	**.026**	2.94	**.007**	-0.38	.740	-0.12	.924	0.26	.853	1.80	8.7	4.21	7.9	7.74	**.006**	-2.42
NET_5	0.75	.521	5.33	9.9	-0.20	8.8	2.84	8.0	2.88	8.1	6.29	**.001**	5.53	**.001**	2.49	.056	2.45	**.039**	-3.03	**.014**	-3.08	**.020**	-0.04	.978	0.86	9.1	4.57	9.1	15.56	**.001**	-3.72

SD: standard deviation. MD: mean difference. |*d*|: Cohen’s-*d* coefficient. HC: healthy control. GD: gambling disorder. SUD: substance use disorder. OB: obesity.

Bold: significant comparison. *p*-values includes Bonferroni-Holm correction.

Results obtained in ANOVA adjusted by the participants’ age and years of education.

## Discussion

This study compares decision making patterns when assessed by the IGT in three different clinical samples (SUD, OB and GD) and a HC. Results display impairments in the overall performance and in the task learning process in all clinical samples when individually compared to the HC.

Results are in agreement with previous studies reporting impaired decision making in SUD and GD individuals [13,14] as well as in individuals with OB[10,12] when independently assessed and compared to a HC group. Additionally, each of the three clinical groups of our sample present more impaired learning across the task than the HC group. Data shows how the HC group presents a preference for the short term wins but after their first set of selections they progressively learn to choose the decks that will lead to bigger long term wins. This is an expected pattern in healthy individuals[4]. In line with previous studies, the decision-making pattern of our HC sample cannot be extended to the clinical groups where learning starts later and/or with a more erratic progress. For instance, OB individuals maintain negative scores until the third set (i.e.: NET_3) where learning starts but to a lesser extent than in the HC group. Previous studies have also reported similar impairments in OB[7,18]. The SUD participants show an earlier learning across the task but improvement progression is slower when compared to the HC ones. The GD individuals display more erratic or random choices. The learning effect is quite small and ends with a score similar to the initial one. Accordingly, previous studies demonstrate how SUD individuals tend to present an adaptive shift in decision-making performance towards the end of the task[19,20] whereas GD individuals do not[21,22]. Therefore, the decision-making impairment of individuals with SUD is probably more associated to a learning delay strategy rather than an inability to learn from the task and this pattern seems not to be extended to GD participants. Finally, no significant differences are found in the overall performance among the clinical groups. All groups achieve low but still positive scores. Results can reflect that to some extent the three clinical samples respond to the punishment cues (although probably to a lesser extent than the HC group) and also that their deficit could be more due to difficulties in reward processing.

Results also display no sex influence among groups. However, some differences are observed when comparing the IGT performance between men and women in the whole sample. Specifically, men present a better performance on the IGT. Previous studies report similar results and suggest that men tend to have a better performance than women in general population[23] and in SUD[24]. Although sex factor was controlled in our study, it would be interesting to explore further in future studies to specifically explore sex differences in the studied samples.

Our data adds to the current literature more evidence of decision making patterns in SUD, OB and GD by comparing the three clinical samples. Importantly, in the analyses conducted none of the differences among the groups could be due to sex or other sociodemographic variables, which adds extra value to the findings reported here. Our study supports the hypothesis that OB shares specific cognitive and neurobiological patterns with SUD and GD which are known to suffer from impairments in dopaminergic pathways that regulate neural systems associated with reward sensitivity and incentive motivation[25]. Accordingly, food addiction has been associated with obesity in previous behavioral and neuroimaging studies[26] and the food intake management difficulties observed in some of the individuals with obesity could be associated with decision making impairments. The clinical implications of these results lie on the identified cognitive patterns that suggest the value of parallel therapeutic approaches among OB, SUD and GD individuals[27,28]. Importantly, the SUD and OB groups display some improvement in decision making; however, they are far from the HC group results (see Fig 1). Hence, cognitive stimulation protocols (through executive function working tools) could potentially benefit these individuals by enhancing their adaptive decision making strategies when treating their disorder. Additionally, some specific differences among the clinical groups could be considered for improving current treatments. For instance, OB individuals take longer to learn the relationship between decks but once they do, their behavior conforms to that of HC. Still, their learning pattern remains less adaptive than the HC one and it should be considered for treatment approaches. Specifically, results may suggest that OB individuals could probably benefit from an early treatment extra emphasis on gaining more adaptive decision making strategies. On the other hand, SUD tend to learn faster than OB individuals and more similar to HC but they display very little improvement when compared to HC. This could be due to more risky behaviors and thus more emphasis should be given to these difficulties. Finally, GD individuals display even more risky behaviors and move faster towards bigger rewards. These difficulties should be targeted in treatment. Future studies should test this hypothesis and further explore these domains in order to (1) help disentangle to what extend these differences are generalizable or constrained to our sample, (2) test the predictive or mediating role of decision making impairments in a study which compares the here studied clinical samples. Furthermore, future studies could further explore sex differences within the studied groups.

The present study has some limitations. Firstly, there are sociodemographic differences concerning education, sex and age across the groups which are representative of the studied disorders. This is an expected result when working with a consecutive clinical sample referred to GD, SUD and OB treatments. However, we have controlled these variables in all the statistical analysis. Although in this study we have paid specific attention to one of the most relevant neuropsychological factors (namely decision making), other cognitive functioning variables or intelligence measures not assessed here may better explain specific differences (more than commonalities) among the clinical groups. Finally, although participants did not present withdrawal symptoms or presented any life time mood-anxiety disorder or mental disorder that could hinder the assessment, the analyses conducted do not explicitly control for the participants’ hunger, anxiety or sadness feelings and these could also play a role in the participants’ performance on the task.

To our knowledge, we present the first study that compares decision making in substance and behavioral related addictions, obese individuals and healthy controls. Results show similar impairments in decision making in the three clinical groups. These impairments are statistically significant when compared to the healthy control group but not different among the three clinical groups. Finally, the clinical groups present significant difficulties in learning across the task when compared to the healthy controls and also some specific differences when comparing clinical groups.
